# Is a single portal venous phase in contrast-enhanced CT sufficient to detect metastases or recurrence in clear cell renal cell carcinoma? – a single-center retrospective study

**DOI:** 10.1186/s40644-022-00444-8

**Published:** 2022-01-21

**Authors:** Florian Hagen, Felix Peisen, Jakob Spogis, Antonia Mair, Konstantin Nikolaou, Arnulf Stenzl, Stephan Kruck, Jens Bedke, Sascha Kaufmann, Wolfgang M. Thaiss

**Affiliations:** 1 Department of Diagnostic and Interventional Radiology, Eberhard-Karls-University, Hoppe-Seyler-Str.3, 72076 Tübingen, Germany; 2 Department of Urology, Eberhard-Karls-University, Hoppe-Seyler-Str.3, 72076 Tübingen, Germany; 3 Clinic of Urology, Siloah St. Trudpert Klinikum, Wilferdinger Str. 67, 75179 Pforzheim, Germany; 4 Department of Urology, University Hospital of Tübingen Eberhard-Karls-University, Hoppe-Seyler-Str.3, 72076 Tübingen, Germany; 5 Diagnostic and Interventional Radiology, Siloah St. Trudpert Klinikum, Wilferdinger Str. 67, 75179 Pforzheim, Germany; 6 Department of Nuclear Medicine, University Hospital Ulm, Albert-Einstein-Allee 23, 89081 Ulm, Germany

**Keywords:** Arterial contrast phase, Image quality, Interreader-agreement, Portal venous contrast phase, Renal cell carcinoma

## Abstract

**Background:**

This study aims at describing the imaging features of the metastatic presentation of clear cell renal cell carcinoma (ccRCC) in arterial (AP) and portal venous phase (PVP) of contrast-enhanced-computed-tomography (CECT) during clinical follow-up (FU) and to evaluate the necessity of a dual phase approach for metastasis detection.

**Methods:**

We identified a total of 584 patients that were diagnosed with ccRCC between January 2016 and April 2020. Inclusion criteria were histologically proven ccRCC with metastatic spread, proven by histology or interim follow-up of at least 2 years and follow-up CT examination with AP and PVP CECT including thorax/abdomen and pelvis. Exclusion criteria were defined by missing or incomplete CT-scans or lack of sufficient follow-up. CT studies of 43 patients with histologically proven ccRCCs were analyzed in retrospect. AP and PVP images were analyzed by two radiologists for metastases, two additional independent radiologists analyzed PVP images only. A 5-point Likert scale was used to evaluate the likelihood off the presence of metastasis. Imaging patterns of the metastases were analyzed visually.

**Results:**

43 patients (16 female; mean age: 67±10 years) with recurrent ccRCC and metastatic disease were included. Three imaging patterns were observed (solid, heterogeneous or cystic metastases), which rarely exhibited calcifications (2%). All metastases showed hyperenhancement in AP and PVP. Inter-reader agreement was substantial (Fleiss’ κ 0.6–0.8, *p*<0.001). No significant differences in sensitivity or specificity between readers (AP and PVP images vs. PVP images only) were present (79.4-85.2%, 97.1-99.6%, p ≥ 0.05). The area under the receiver-operating-characteristic (ROC) curve was between 0.901and 0.922 for all four radiologists.

**Conclusions:**

Similar rates for detection, sensitivity and specificity of metastasis and local recurrence in ccRCC were observed irrespective of using a dual-phase protocol with AP and PVP or a single PVP protocol only. Thus, a single-phase examination of PVP can be sufficient for experienced radiologists to detect metastatic disease in the follow-up of ccRCC patients.

## Introduction

With an incidence of about 115 per 100.000 habitants in Europe renal cell carcinoma (RCC) still leads to a considerable number of deaths each year (49 per 100.000) [1]. However, with the introduction of new immune checkpoint inhibitors the progression-free survival as well as the overall survival has increased over the last years, leading to a higher rate of follow-up examinations per patient [2]. Besides chromophobe and papillary RCCs, clear cell RCC (ccRCC) represent the most common histological subtype, differing in metastatic predilection sites, among other features [3]. Detection and staging of RCCs by cross-sectional imaging achieves a good sensitivity and specificity, both by computed tomography (CT) and by magnetic resonance imaging (MRI) [4]. Therefore, an established and approved staging recommendation regimen is essential. Guidelines of the European Association of Urology (EAU) define criteria for initial diagnosis and staging of renal cell carcinoma by the employment of a multi-phasic contrast-enhanced computed tomography (CECT) of the abdomen and chest [5]. However, imaging protocols for an adequate follow-up examination are still up for debate [6–9].

Metastatic lesions of ccRCC occur mainly in the lungs and bones [10]. While local recurrences, pancreatic or hepatic lesions are less common [10], a decreased contrast between normal parenchyma and the metastases in parenchymal organs can be detrimential for the detection of metastases in solid organs [11–13]. Thus, some authors favor the addition of an arterial phase (AP) to a portal venous phase (PVP) for staging in the follow-up of ccRCC [11, 12, 14]. This is, however, in contrast to ongoing attempts to lower radiation doses and examination time while maintaining diagnostic performance. These trade-offs seem to be tolerable to some extend as not every single metastasis necessarily leads to a change in the therapeutic regimen [12].

Therefore, the aim of this study was to evaluate the impact of a two-phase contrast-enhanced CT of the abdomen and chest in patients with metastatic ccRCC in comparison to a single, portal venous phase CT, regarding the diagnostic accuracy for the detection of recurrent tumors or metastases.

## Materials and methods

### Data collection and study cohort

**Fig. 1:**
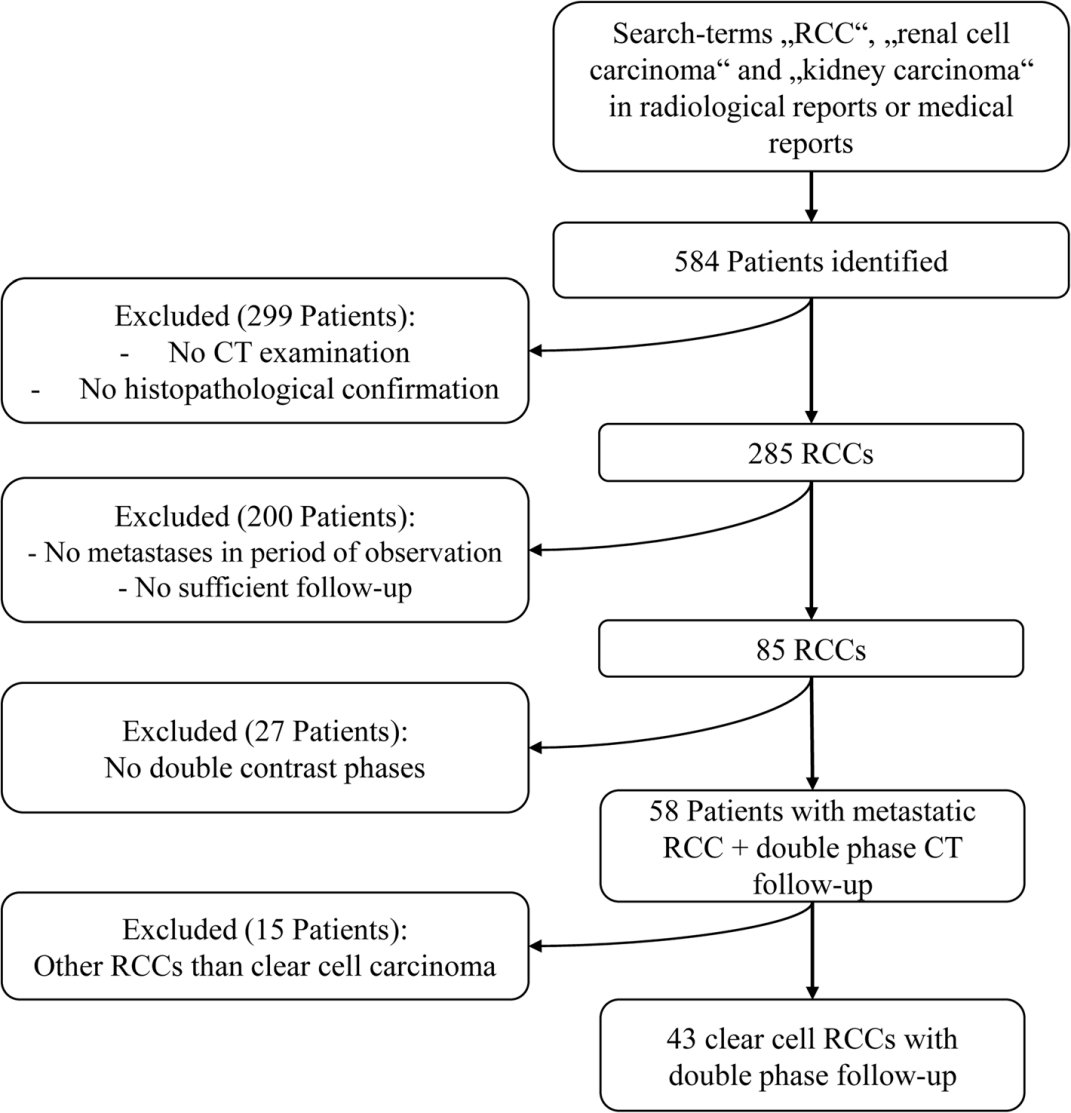
Study population

This retrospective data evaluation was approved by the institutional review board (approval number 197/2020BO2). Verbal and written informed consent were waived for this retrospective study. The data were collected from the institution’s electronic medical records. We identified a total of 584 patients that were diagnosed with ccRCC between January 2016 and April 2020. Inclusion criteria were as follows: (1) Histologically proven ccRCC, (2) metastatic spread of the ccRCC, proven by histology or interim follow-up of at least 2 years as evidence of metastasis, (3) follow-up CT examination with AP and PVP CECT. Exclusion criteria were defined by missing or incomplete CT-scans or an insufficient follow-up as evidence of new metastatic occurrences. This was most frequently due to the contraindication of contrast agent injection such as a creatinine clearance below 30mL/min/kg resulting in a change of the follow-up protocol (non-enhanced chest CT and abdominal MRI). Of those patients remaining, 43 were included in the study (Fig. 1). Of those, 22 patients had no previous treatment, 11 patients had undergone first-line treatment (6 with checkpoint inhibitor/5 with tyrosine kinase inhibitor) and 10 had undergone second line treatment (6 with checkpoint inhibitor/4 with tyrosine kinase inhibitor).

### Image acquisition

All CT examinations were performed on a third generation dual-energy scanner consisting of two 192 detector rows (Siemens SOMATOM Force, Siemens Healthineers, Forchheim, Germany). The CT-images of the thorax and abdomen/pelvis were acquired in dual-energy technique using a tube current of 300mAs (CARE Dose4D) for tube A (100 kV) and ref. 232mAs tube current for tube B (Sn150kV), 0.6 mm single collimation width, spiral pitch factor 0.6, matrix 512 × 512 and a convolution kernel Br40 for arterial and Bf40 for PVP image data sets (see Table 1). Image reconstruction was performed with a thickness of 3 mm in 2 planes (axial and coronary) for both contrast phases. Furthermore, high-resolution reconstructions and maximum intensity projections from the PV image datasets were included in the analysis. All patients received Iomeprol as contrast agent (Imerone 400; Bracco, Milan, Italy) intravenously by a dual syringe injector at 2-3 mL/sec (CT Stellant, Medrad, Indianola, PA, USA) followed by a saline chaser bolus. To improve both image quality and parenchymal enhancement the contrast agent was applied taking into account a lean body weight-adapted dosing protocol [15] (body weight in g + 20 = amount of contrast agent in mL.) following the subsequent flow and delay depending on the contrast phase (see Table 1): Based on the findings of Itoh et al., reporting similar detection rates for the late arterial contrast phase after 24.6 to 36.0 Sect.  [16]. The portal venous phase was set based on the findings of Birnbaum et al., reporting nephrographic phase after 60 to 136 Sect.  [17].

**Table 1:** Image acquisition parameters and contrast medium

	Arterial Phase	Portal Venous Phase
Single collimation width	0.6	0.6
Spiral pitch factor	0.6	0.6
Tube voltages (keV)	120	100 and 150
Ref. tube current (mAs)*	275	300 and 232
Matrix	512 × 512	512 × 512
Reconstruction kernel	Br40	Bf40
Delay between contrast agent injection and scan	35 s p.i.	65 s p.i.
Flow	2-3 mL/sec	2-3 mL/sec

*Care Dose4D, p.i.: post injection

### Assessment of imaging features on CT

Four radiologists with 2, 4, 8 and 12 years of experience in reading oncological CT images analyzed the data sets in two groups and were blinded to the number and location of the metastases: The first group consisted of the less experienced radiologists (2 and 4 years of experience) and read both data sets, the AP and the PVP. The second group of radiologists (8 and 12 years of experience) only had the PVP data sets available for the identification of metastases. As ground truth for the presence of metastatic lesions every patient had a follow-up within a timespan of at least 2 years with interval growth or the metastasis were resected and proven by histology. Suspicious lesions were defined as hypervascularized lesions with distortion of the normal organ structure. Ten potential manifestation regions were defined before reading: either local recurrences, or systemic recurrences, such as manifestation at the opposite kidney, peritoneal metastases, lymph node metastases, pancreatic metastases, hepatic metastases, adrenal metastases, pulmonary metastases, soft-tissue metastases and bone metastases. All lesions were scored individually on the 5-point Likert scale (1 = no metastasis, 2 = unlikely metastasis, 3 = possible metastasis, 4 = most likely metastasis, 5 = definite metastasis). Only lesions classified as “most likely” or “definite” (Likert 4 and 5) were considered as positive metastasis. Furthermore, the lesions were classified depending on their visual aspect and the measured Hounsfield units as being solid, cystic heterogeneous. The presence or absence of calcification was also determined.

### Statistics

Statistical analysis was performed using SPSS (version 27.0.0, IBM, Armonk, NY, USA). Continuous data were expressed as means ± standard deviation (SD). Differences in the number of imaging patterns (solid, cystic, heterogenous) were tested by the Kruskal–Wallis H test. Interrater reliability between the 4 readers was tested with Fleiss’ Kappa (κ). Values from 0.0 to 0.2 indicate slight agreement, 0.21 to 0.40 fair agreement, 0.41 to 0.60 moderate agreement, 0.61 to 0.80 substantial agreement, and values ranging from 0.81 to 1.0 indicate almost perfect or perfect agreement [18]. Sensitivity, specificity as well as accuracy were calculated for every radiologist and plotted on a receiver operating characteristics (ROC)-curve. Determination of the optimal cut-off point was achieved by the Youden index. After verification of the non-Gaussian distribution of every parameter by the Shapiro-Wilk test, we opted for a non-parametric test (Kruskal-Wallis-H test) to analyze differences between the four independent radiologists. A two-tailed p-value of less than 0.05 was considered to indicate statistical significance.

## Results

**Fig. 2:**
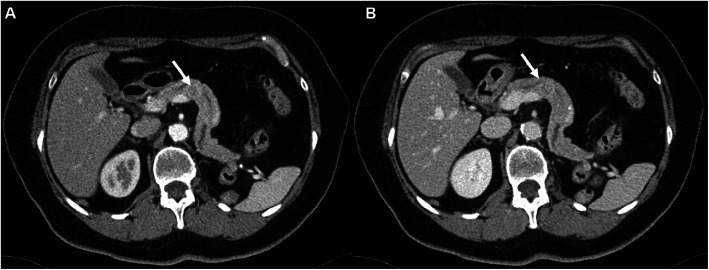
Axial CT images of the pancreas in arterial (**A**) and portal venous (**B**) phases of a 65-year-old woman with metastatic RCC. In both phases the metastasis is visible as a hypervascularized lesion (white arrows)

The mean patient age at the time point of CT examination was 67 ± 10 years. The 27 male and 16 female patients presented with a total of 155 metastatic lesions (mean 3.6 metastatic lesions per patient). The majority of lesions were solid lesions (102/155) followed by heterogeneous (43/155) and cystic lesions (10/155) (*p*=0.001). Only 2% of the lesions presented with calcifications. Most of the metastatic lesions were found in the lungs (*n* = 31, 72.1%) followed by lymph node metastases (*n* = 30, 69.8%) and bone metastases, appearing osteolytic with a soft tissue component (*n* = 18, 41.9%). Metastases were found less frequently in the pancreas (*n* = 7, 16.3%, example given in Fig. 2), soft tissues (*n* = 8, 18.6%) and the contralateral kidney (*n* = 9, 20.9%).

Twenty-one patients received a therapy with an immune-checkpoint-inhibitor, a tyrosine-kinase-inhibitor at follow-up examination (Table 2). Of those, 10 patients presented with a second line therapy (e.g. Nivolumab) due to their initial synchronously metastatic ccRCC.

**Table 2:** Patient characteristics

	Total
Age [mean ± standard deviation]	67 ± 10 years
Sex [n]	27 male and 16 female
No treatment [n]	22 (51.2%)
Treatment with Checkpoint inhibitor (Nivolumab, Pembrolizumab+ Axitinib) [n]	12 (27.9%)
Treatment with tyrosine kinase inhibitor (Sorafenib, Cabozantinib, Pazopanib, Sunitinib) [n]	9 (20.9%)
Metastatic lesions divided by organ systems (total) [n]	155
Pulmonary [n]	31 (20.0%)
Lymphatic [n]	30 (19.4%)
Osseous [n]	18 (11.6%)
Hepatic [n]	16 (10.3%)
Adrenal [n]	13 (8.4%)
Peritoneal [n]	12 (7.7%)
Local [n]	11 (7.1%)
Contralateral kidney [n]	9 (5.8%)
Soft tissue [n]	8 (5.2%)
Pancreatic [n]	7 (4.5%)

**Fig. 3:**
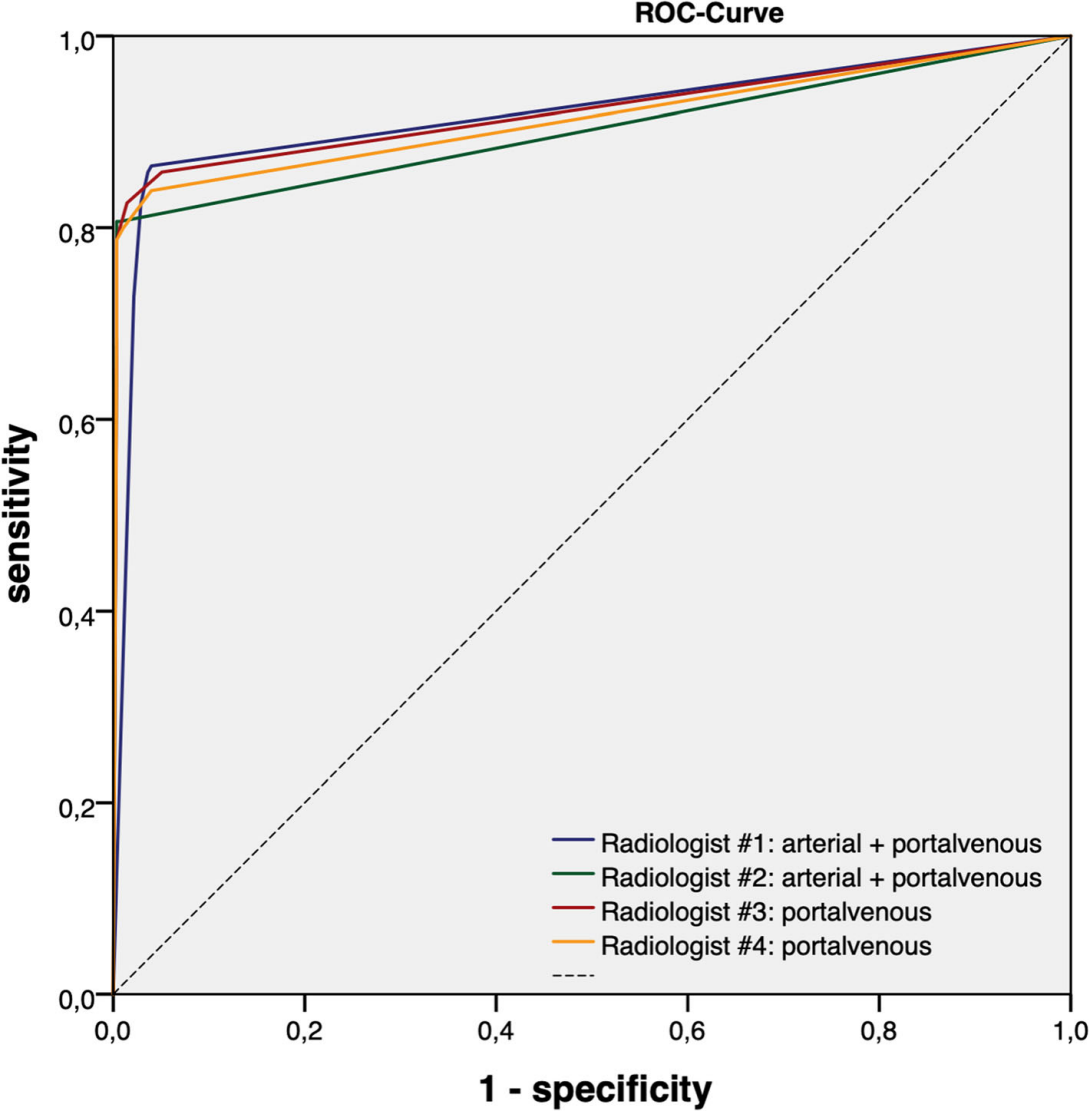
Receiver operating characteristic (ROC) curves depending on the image protocol

No significant difference in sensitivity, specificity and accuracy between group 1 (Radiologist #1 and #2, reading AP and PVP) and group 2 (Radiologist #3 and #4 reading PVP only) could be found (p ≥ 0.05, Table 3). Overall, a sensitivity of 82.3% for the first and 83.1% for the second group was achieved. The area under the receiver operating characteristic (ROC) curve ranged between 0.901 and 0.922 (CI: 0.884 – 0.959, Table 4; Fig. 3).

**Table 3:** Sensitivity, specificity and accuracy of the 4 different radiologists depending on the image data sets

Radiologist #1 AP + PVP	Accuracy	92.8%
	Sensitivity	85.2%
	Specificity	97.1%
Radiologist #2 AP + PVP	Accuracy	94.9%
	Sensitivity	79.4%
	Specificity	99.6%
Radiologist #3 PVP	Accuracy	93.7%
	Sensitivity	83.2%
	Specificity	99.6%
Radiologist #4 PVP	Accuracy	93.5%
	Sensitivity	83.1%
	Specificity	99.3%

**Table 4:** Area under the ROC-Curve

	Radiologist #1 (AP+PVP)	Radiologist #2 (AP+PVP)	Radiologist #3 (PVP only)	Radiologist #4 (PVP only)
AUC	0.918 (CI: 0.884 – 0.952)	0.901 (CI: 0.863 – 0.939)	0.922 (CI: 0.889 – 0.956)	0.913 (CI: 0.878 – 0.949)

CI: confidence interval

**Fig. 4:**
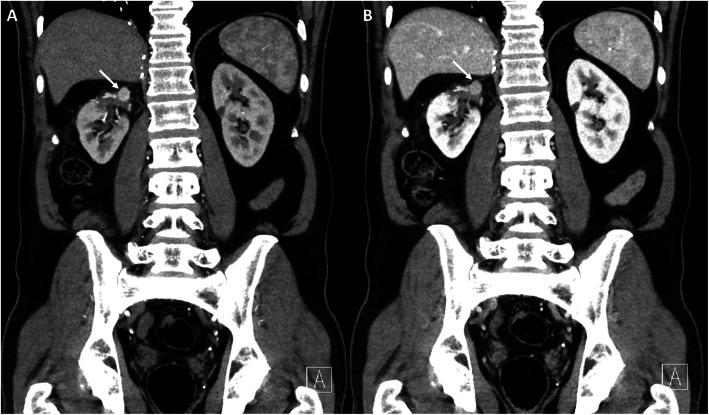
Coronal CT images of the local recurrence in arterial (**A**) and portal venous (**B**) phases of a 64-year-old man with multiple metastatic RCC

Overall, the four radiologists had a good to very good inter-reader agreement (κ ranging from 0.6 to 0.83, *p*<0.001). Pulmonary metastases were associated with the worst inter-reader agreement whereas peritoneal metastases coincided with the best inter-reader agreement. Lesions in parenchymatous organs as for example pancreatic lesions (Fig. 2) or local recurrences (Fig. 4) achieved an average interrater agreement. No significant difference (p ≥ 0.05) in total lesion detection could be registered between the four radiologists (118 to 136 of 155 possible lesions detected, Table 5).

**Table 5:** Inter-reader agreement depending on metastatic lesions

	Radiologist #1	Radiologist #2	Radiologist #3	Radiologist #4	Fleiss‘ Kappa κ	p-value
Pulmonary metastases [n]	31	24	27	27	0.60 ± 0.05	<0.001
Lymph node metastases [n]	26	20	24	23	0.61 ± 0.05	<0.001
Osseous metastases [n]	16	16	17	16	0.76 ± 0.05	<0.001
Hepatic metastases [n]	13	14	15	15	0.66 ± 0.05	<0.001
Adrenal metastases [n]	12	8	6	7	0.63 ± 0.05	<0.001
Peritoneal metastases [n]	9	8	8	8	0.83 ± 0.06	<0.001
Local recurrences [n]	10	10	9	9	0.69 ± 0.05	<0.001
Contralateral kidney [n]	6	6	5	5	0.76 ± 0.04	<0.001
Soft tissue metastases [n]	7	6	8	7	0.69 ± 0.05	<0.001
Pancreatic metastases [n]	6	6	6	6	0.66 ± 0.05	<0.001

## Discussion

This study shows that the detection of metastatic lesions of ccRCC can be performed with excellent comparable sensitivity and specificity between single PVP imaging and dual, AP and PVP, imaging.

Both groups achieved a good sensitivity of 82.3% for the AP + PVP group and 83.1% for the PVP only group, whereas the specificity of both groups was excellent with 98.4% and 99.5%, respectively. This resulted in a good to very good interrater agreement for the ten potential metastatic sides evaluated in this study. Moreover, all four readers achieved a very good area under the ROC curves (>0.9, Table 4).

In our cohort, 7 of the 43 patients had pancreatic metastases. Of these, every single radiologist detected 6 (86%) pancreatic lesions as such. Recent publications claimed that especially metastatic lesions of the pancreas are more easily detectable in the arterial contrast phase [11, 13]. This might be due to the early arterial enhancement of pancreatic metastasis compared to parenchyma [19]. Particularly, initial small sized pancreatic metastases, which often occur metachronous several years after the initial diagnosis, might increase true-positive lesion detection [20]. In fact, even the radiologists who read the PVP datasets only achieved a higher detection rate than in recently published literature (86% vs. 50 – 69%) [11]. A reason for this might be the earlier imaging time point in our study protocol compared to Corwin et al. (65 s vs. 80-90 s) [11]. In contrast to Jain et al., who reported a visualization of pancreatic metastases in the PVP or AP datasets only of up to 25% [12], we perceived all pancreatic metastases equally in both phases. Furthermore, 4 of the 7 pancreatic metastases occurred in patients receiving no therapy. Especially targeted-therapy results in reduced density of parenchymatous organs in the arterial phase of CECT [21]. These might be reasons for the contrast between pancreatic parenchyma and pancreatic metastasis (see Fig. 2).

Furthermore, good interrater agreement between the use of PVP only and the combination of PVP and AP was achieved for the upper abdominal organs, especially for detection of hepatic and renal metastases. Our results reinforce the assumption that an additional AP in the detection of ccRCC metastasis might be of limited value that has been controversially discussed in the literature for liver metastases [12, 22]. Advances in DECT with an improved assessment of hypodense liver lesions in the PVP by switching keV levels in monoenergetic reconstructions [23] might confirm this notion in the future. Some authors state the necessity of an AP for the detection of renal metastases, especially for local recurrence [14]. For our cohort, we cannot confirm this hypothesis as we found that local recurrences were detected at comparable rates in the PVP only (10 lesions detected in the AP+PVP group vs. 9 lesions detected for PVP only group, Table 5). Griffin et al. reported that local recurrences can be shown on CECT as solid enhancing masses [14]. As the contrast is higher in hypervascularized lesions in the AP compared to the PVP [12], Raptopoulos et al. proposed a practical approach by considering an AP und PVP for initial evaluations of patients with metastatic RCCs and the use of the PVP only for follow-up examinations [24].

Pulmonary lesions are the most common site of recurrence in ccRCC [10, 13], which is in line with 72.1% of lung metastases in our cohort. Incidental pulmonary nodules are commonly seen on CT-scans of the chest and therefore the diagnosis of ccRCC metastasis might be difficult without previous examinations [25]. With only one examination in our study, interrater agreement between the 4 radiologists was still decent (κ 0.6, Table 4). As reported by Price et al., pulmonary metastases most often appear as solid nodules and masses, sometimes surrounded by a peripheral ground-glass halo [26]. However, adding more contrast phases would not lead to additional information for the characterization of small nodules.

In our study cohort, the missed lesions would not have led to treatment changes as there were more than 3 metastatic lesions present per patient. This is in line with findings reported by Jain et al. where only 2% of the metastatic lesions lead to a different treatment [12]. However, this might be of importance when only a limited number of metastases are present as the patients with oligometastatic disease are prone to metastasectomy instead of systemic treatment. There were no significant differences when comparing the specificity between the two groups in our study (98.4% vs. 99.6%).

Three typical patterns of the metastases could be differentiated: solid, heterogeneous and cystic. As reported by Smith et al., targeted therapies result in distinct patterns of metastatic ccRCCs as they might change their appearance and enhancement pattern from an initially homogeneous and non-enhancing mass [27]. This is even more important as contemporaneous reduction in tumor size and attenuation were correlated with favorable clinical outcomes [28]. However, while no different imaging response criteria are defined for assessing therapy response diameter-based criteria remain essential [29].

We conclude that staging of metastatic ccRCCs using PVP only resulted in comparable results to dual-phase CECT and help to reduce radiation exposure. Taking the number of repetitive staging examinations into account the radiation exposure can be substantially reduced, although the 5-year overall survival in metastatic ccRCC patients is limited [30, 31].

### Limitations

This single-center study is limited by its nonrandomized and retrospective design. Moreover, the lack of histological confirmation of metastases had to be compensated by successional CT-scans permitting the evaluation of tissue dynamics. Another confounder may be the fact, that about half of the patients were currently receiving therapy, which is likely to cause altered perfusion characteristics of the metastases [12]. As a result of the low number of cases included, the data may have overestimated the potential of the portal venous contrast enhanced phase for the detection of metastases. Furthermore, the two “less experienced” radiologists had both phases whereas the two experienced radiologists only had the PV phase, resulting in a certain degree of inter-reader bias. However, by getting comparable detection rates in both groups the experience seems to replace an additional AP.

## Conclusions

In this study, we demonstrate similar rates for detection, sensitivity and specificity of metastases and local recurrence of ccRCC when comparing a dual phase protocol with arterial and portal-venous contrast to a single-phase protocol with portal-venous contrast. Considering a certain experience level, we conclude that a single-phase examination might be sufficient for follow-up examinations in patients with metastatic ccRCC.

## Data Availability

All data generated or analysed during this study are included in this published article.

## Abbreviations

AP: Arterial phase
ccRCC: Clear cell renal cell carcinoma
CECT: Contrast-enhanced computed tomography
CT: Computed tomography
EAU: European Association of Urology
FU: Follow-up
MRI: Magnetic resonance imaging
PVP: Portal venous phase
RCC: Renal cell carcinoma
ROC: Receiver operating characteristics
SD: Standard deviation
